# An invitation to recent graduates: publish your dissertation/thesis background section as a review in Virology Journal

**DOI:** 10.1186/1743-422X-4-46

**Published:** 2007-06-02

**Authors:** Robert F Garry

**Affiliations:** 1 Department of Microbiology & Immunology Tulane University Health Sciences Center, Tulane, New Orleans, LA, USA

## Abstract

Virology Journal will publish background sections of approved dissertations or theses as Review Articles.

## Background

May is graduation time. Over the years I have observed that a large amount of graduate student labor is spent on producing a comprehensive background section for dissertations or theses. Often, only the student and their committee (perhaps also some proud parents) are the only ones who ever read or benefit from this effort. Virology Journal will provide a mechanism for timely dissemination of this valuable information to the broader scientific community by publishing these background sections as Review Articles. Peer review will be expedited based on the notion that this background section will have already been stringently reviewed by a university committee. There are a few stipulations:

1. The review should relate principally to viruses, prions or similar infectious agents.

2. The dissertation/thesis has been approved by a Master's or Doctoral university committee, and all requirements for the degree have been met.

3. The background section was written not more than two years ago. This requirement is in part to accommodate recent graduates, especially those of Katrina-affected institutions. In the future this requirement will be changed to not more than one year out-of-date.

4. This review is a single-author publication. The author's mentor and dissertation/thesis advising committee must be listed by name in the acknowledgement section. Virology Journal considers multi-authored reviews, but these are subject to the normal course of external peer review.

5. The penultimate section should be a brief summary of the experimental results obtained in the dissertation/thesis appropriately referenced.

6. The final section should be a brief biographical summary of the author.

7. Journal formatting must be followed, including references.

8. As in all reviews, all permissions for figures published elsewhere must be obtained.

9. Article fees will follow standard BMC policy. Fee waivers will be considered on a case-by-case basis.

Please feel free to contact me directly, if you require any additional information about this new Virology Journal feature.

## Competing interests

I am the Editor-in-Chief of Virology Journal.

## Authors' contributions

I wrote this article. Several graduate students and colleagues have suggested similar mechanisms over the years.

